# Self-Rated Health in Older Adults (60+) Who Are Sexually Active: Association With Pleasure

**DOI:** 10.1093/geroni/igaa057.1001

**Published:** 2020-12-16

**Authors:** Nicole Viviano

**Affiliations:** University of Maryland, Baltimore, Gaithersburg, Maryland, United States

## Abstract

Older adults who engage in sexual activity (SA) tend to have greater enjoyment in life compared to their less sexually active counterparts. Further, older adults tend to report more satisfying or pleasurable SA when less health conditions are reported. Although self-rated health (SRH) is often incorporated in studies concerning SA, it is not always included when examining sexual intimacy, pleasure, and pain/discomfort. It is important to understand how SRH is associated with both SA and sexual pleasure as it could indicate when providers need to initiate conversations with their patients regarding safe sex practices and/or diagnose related health conditions (i.e. erectile dysfunction, vaginal dryness, etc.). The current study examined the association between SRH and experiencing pleasurable sexual interactions in older adults (60+). The Midlife in the United States (MIDUS) Refresher dataset (2011-2014) was used for this secondary data analysis. The dependent variable is pleasure in sexual interaction; the independent variable is self-rated health. Covariates include marital status, race, sex, sexual intimacy and sexual pain/discomfort. An ordinal logistic regression shows that for every one-unit increase in SRH, there is 1.341 times odds of experiencing pleasure in sexual interactions in older adults 60+ after adjusting for covariates. Individuals who had higher SRH had increased odds of having pleasurable sexual encounters. It is already clear that older adults are engaging in sexual activity, despite stereotypical myths. Further understanding the relationships between factors of SA like pleasure, pain/discomfort, and intimacy and health factors might allow for the extension of a healthy sexual life.

